# Diagnosis and Management of Heart Failure in Elderly Patients from Hospital Admission to Discharge: Position Paper

**DOI:** 10.3390/jcm10163519

**Published:** 2021-08-10

**Authors:** Thibaud Damy, Tahar Chouihed, Nicholas Delarche, Gilles Berrut, Patrice Cacoub, Patrick Henry, Nicholas Lamblin, Emmanuel Andrès, Olivier Hanon

**Affiliations:** 1 Service de Cardiologie, CHU H. Mondor, 94000 Créteil, France; 2 Service des SAMU-SMUR-Urgences, Centre d’Investigations Cliniques-1433, INSERM UMR_S 1116, Université de Lorraine, CHRU Nancy, F-CRIN INI-CRCT, 541000 Nancy, France; t.chouihed@chru-nancy.fr; 3 Service de Cardiologie, CH Pau, 64000 Pau, France; n.delarche@wanadoo.fr; 4 CHU Nantes, Pôle Hospitalo-Universitiare de Gérontologie Clinique, 44000 Nantes, France; gilles.berrut@chu-nantes.fr; 5 Groupe Hospitalier Pitié-Salpêtrière, Department of Internal Medicine and Clinical Immunology, AP-HP, 75000 Paris, France; patrice.cacoub@gmail.com; 6 Service de Cardiologie, APHP, Hôpital Lariboisière, 75000 Paris, France; patrick.henry@aphp.fr; 7 Service de Cardiologie, Institut Pasteur de Lille, CHU de Lille, Université de Lille, U1167, 59000 Lille, France; nicolas.lamblin@chru-lille.fr; 8 Service Méd. Interne, Diabète, Maladies Métaboliques, Clinique Médicale B, CHU Strasbourg, 67000 Strasbourg, France; emmanuel.andres@chru-strasbourg.fr; 9 Service de Gériatrie, APHP, Hôpital Broca, Université de Paris, 54 Rue Pascal, 75013 Paris, France; olivier.hanon@aphp.fr

**Keywords:** practical guidance, heart failure management, elderly

## Abstract

Multidisciplinary management of worsening heart failure (HF) in the elderly improves survival. To ensure patients have access to adequate care, the current HF and French health authority guidelines advise establishing a clearly defined HF patient pathway. This pathway involves coordinating multiple disciplines to manage decompensating HF. Yet, recent registry data indicate that insufficient numbers of patients receive specialised cardiology care, which increases the risk of rehospitalisation and mortality. The patient pathway in France involves three key stages: presentation with decompensated HF, stabilisation within a hospital setting and transitional care back out into the community. In each of these three phases, HF diagnosis, severity and precipitating factors need to be promptly identified and managed. This is particularly pertinent in older, frail patients who may present with atypical symptoms or coexisting comorbidities and for whom geriatric evaluation may be needed or specific geriatric syndrome management implemented. In the transition phase, multi-professional post-discharge management must be coordinated with community health care professionals. When the patient is discharged, HF medication must be optimised, and patients educated about self-care and monitoring symptoms. This review provides practical guidance to clinicians managing worsening HF in the elderly.

## 1. Introduction

In France, heart failure (HF) affects more than a million people each year, of whom 75% are aged 75 years or over [1]. This elderly population represents approximately half of the potentially avoidable hospitalisations in that age group, at a cost of almost 2.8 billion Euros in 2017 [2].

Patient pathways for acute heart failure (AHF) usually depend on symptom severity. Normally, patients with acute symptoms call an ambulance or go directly to the hospital Emergency Department (ED). However, patients with symptoms lasting longer than several weeks tend to consult a general practitioner (GP) or cardiologist who may refer them to a cardiac ward or cardiac intensive care unit (CICU). However, the availability of specialist cardiology consultations and beds is limited so few patients are treated on cardiac wards [3,4]. The French heart failure observational survey found that although 77% of elderly patients arrived at the cardiology department or the CICU, 18% were then transferred to an internal medicine ward [5]. Besides the benefit of specialised cardiac treatment, multidisciplinary health care teams are also important in the management of HF and associated comorbidities [3], and health authorities now encourage multidisciplinary assessment and coordination for HF [5,6,7]. 

Yet, as access to cardiologists in France is insufficient, recommended HF treatments are underutilised and left ventricular ejection fraction (LVEF) has been found to be available for only 52% of hospitalised geriatric patients, which is particularly problematic in this population [8]. Furthermore, the elderly often have comorbidities, including depression, cognitive disorders, delirium, malnutrition, sarcopenia, falls, and loss of autonomy that can delay HF diagnosis, contribute to frailty, trigger decompensation, have functional consequences and ultimately alter prognosis [4]. 

In France, about 25% of HF patients are readmitted within thirty days of discharge, [9] this may largely be a result of inadequate treatment prescription after discharge or poor adherence to prescribed treatment [10]. The mortality risk for these readmitted HF patients can be as high as 10% [11]. Yet, only one third readmitted HF patients are seen by a cardiologist when readmitted [12]. 

**Figure 1:**
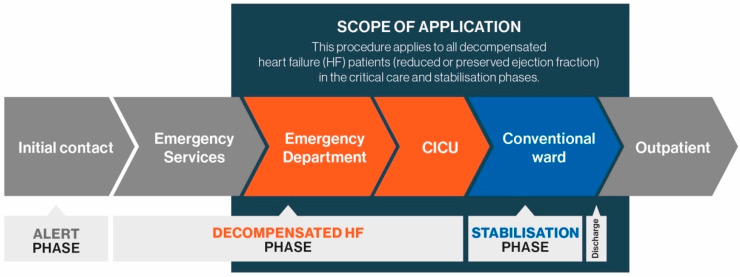
Patient pathway.

This practical review aims to provide up-to-date information for French clinicians treating elderly HF patients with varying aetiology, focusing on the decompensated HF and stabilisation phases of the care pathway (Figure 1), and forming part of a French initiative that aims to promote better healthcare organisation and improve cooperation between healthcare professionals for the benefit of patients. We hope this information will help combat therapeutic inertia, optimise treatment, and prevent avoidable hospital readmissions [13].

An expert working group, including clinicians specialised in cardiology, geriatrics, internal medicine, and emergency medicine, used clinician experience, local and European research, and guidelines to create this position paper. The paper also includes practical recommendations that can be adapted to various hospital settings. 

## 2. Patient Care Pathway

### 2.1. Pre-Screening and HF Diagnosis in the Emergency Department

A potential heart failure (HF) candidate is any patient who presents with Shortness of breath, either in a supine position or on exertion, significant Weight gain over several days, lower limb Oedema and Tiredness following minimal exertion with reduced activity levels and worsening muscle loss (or SWOT) [14]. The diagnostic workup must be started immediately to initiate appropriate treatment and management [15].

HF diagnosis is based on left ventricular ejection fraction (LVEF) and varies from patients with preserved LVEF (≥50%) (HFpEF), mid-range LVEF between 40% and 49% to those with reduced LVEF (<40%) (HFrEF). HFpEF occurs more often in older, female patients with pre-existing hypertension and atrial fibrillation (AF). Diagnosing HFpEF is particularly challenging as most have left ventricular wall thickening or increased atrial size due to elevated filling pressure. 

When HF is suspected, other non-cardiovascular differential diagnoses such as life-threatening clinical conditions, pulmonary infection, severe anaemia, acute renal failure, as well as precipitating factors must be identified and managed [16].

Several diagnostic scores are useful to refine the diagnosis. The scale derived from the PREDICA study estimates AHF probability on a scale from 0 (unlikely) to 10 (certainty) using eight predictive AHF factors in patients with acute dyspnoea in emergency, including age >80 years, >three cardiovascular medications, paroxysmal dyspnoea, jugular vein distension or hepatojugular reflux, lower limb oedema, crackling rales, no bronchodilator treatment, and abnormal ECG [15].

The validated 11-point Brest score [17] helps emergency clinicians predict chronic heart failure) CHF among patients presenting with dyspnoea. One point is allocated for each of the following variables: age >65 years, patient history (nocturnal onset, sudden dyspnoea, orthopnoea), risk factor (prior episodes of CHF, myocardial infarction (MI), chronic pulmonary disease), clinical findings (pitting leg oedema, pulmonary crackles), and abnormal ECG (atrial fibrillation/flutter, ST-segment abnormalities). The score indicates if the probability of CHF is low (0–3 points), intermediate (4–8 points) or high (>9 points). 

### 2.2. Stratify Severity

Although the correlation between symptom severity and left ventricular function is poor, symptom severity is clearly related to survival. Importantly, patients presenting mild symptoms may nevertheless have cardiopulmonary instability and be at increased risk of hospitalisation and death [18,19,20].

Therefore, it is important to determine the cardiopulmonary severity using objective measurements for: [9].

- Dyspnoea severity: respiratory rate, intolerance of the supine position, breathing effort, and oxygen saturation.
- Abnormal blood pressure (BP): systolic and diastolic blood pressure (within the context of the patient’s history).
- Abnormal heart rate (HR) and rhythm (within the context of the patient’s history).
- Other cardiopulmonary instability signs including body temperature and signs/symptoms of hypoperfusion (cool extremities, narrow pulse pressure, mental status) [9].

Once cardiopulmonary severity is established, the patient can be triaged accordingly. In an ED, the FRENCH (French Emergency Nurses Classification in Hospital) scale is useful to triage patients from (5) the least to (1) the most urgent [21]. 

Alternatively, precipitating factors causing decompensation requiring urgent correction can be prioritised, including acute coronary syndrome, hypertensive emergency, rapid arrhythmia, severe bradycardia, conduction disturbance, acute mechanical cause, or acute pulmonary embolism (PE) [16].

### 2.3. Specificity of Diagnosis in Older Patients

In the elderly, HF often occurs with both cardiovascular and non-cardiovascular comorbidities [22]. Concomitant diseases (e.g., respiratory disease, anaemia, exertional disability) make interpreting dyspnoea difficult, and peripheral oedema may be observed in other common conditions (malnutrition, venous insufficiency, lymphoedema, renal diseases).

Moreover, HF should be suspected with atypical symptoms such as delirium, agitation, and confusion, especially in patients with dementia. Sarcopenia, falls and malnutrition are common in older HF patients and represent poor prognostic factors, requiring specific geriatric management [4]. 

## 3. Initial Assessment and Differential Diagnoses 

History, clinical examination and ECG.

**Figure 2:**
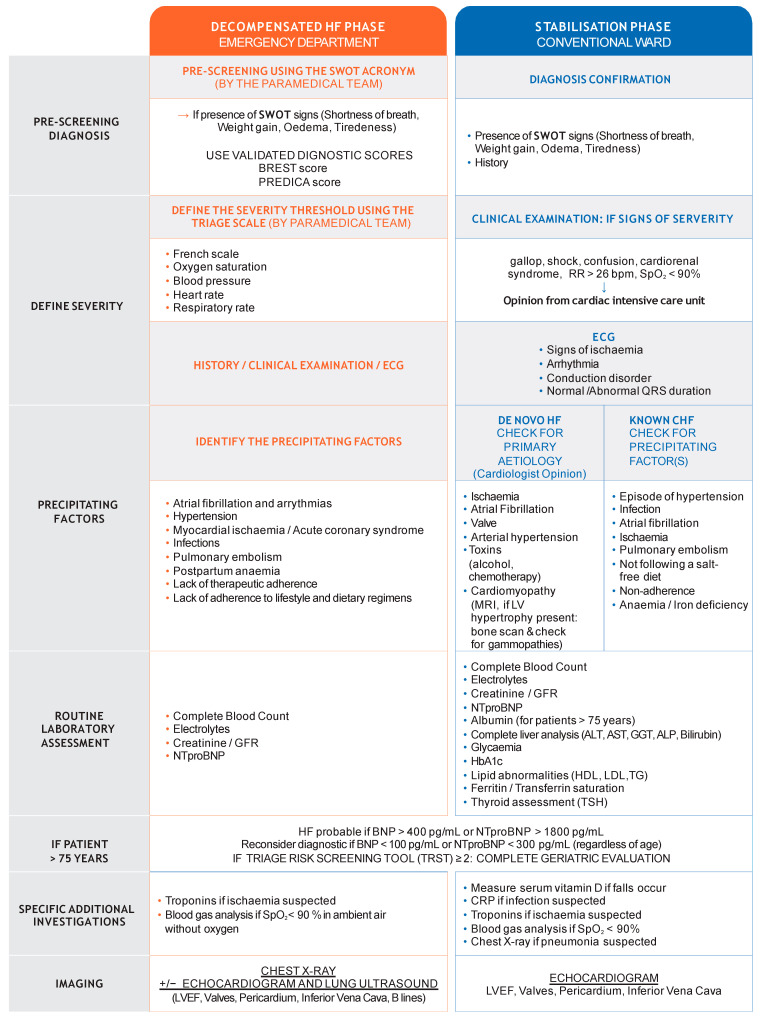
Initial diagnosis. This figure was developed using expert consensus, guidelines and recommendations [7,9,14,16,24,25,26,27] and specific studies [15,17,21,28].

ECGs are rarely normal in acute heart failure (AHF), they indicate underlying cardiac disease and potential precipitating factors [16] and can exclude ST-segment elevation MI [9]. (Figure 2).

### 3.1. Laboratory and Clinical Assessments 

A routine blood test and natriuretic peptide (NP) measurement is essential for all patients with acute dyspnoea and suspected AHF to differentiate from non-cardiac causes [16]. Notably, the upper threshold value of NTproBNP increases with age. Albumin should be measured in patients aged over 75 years to check for malnutrition. 

Imaging is a useful initial assessment. Although 20% of patients with AHF have an apparently normal chest X-ray, pulmonary venous congestion, pleural effusion, interstitial or alveolar oedema and cardiomegaly can be identified [16]. Chest X-rays can also identify non-cardiac diseases such as pneumonia or non-consolidated pulmonary infections which may contribute to symptoms [16].

Immediate echocardiography is only essential in haemodynamically unstable patients and patients with acute life-threatening structural or functional cardiac abnormalities such as aortic dissection or acute valvular regurgitation [16]. However, echocardiography is needed once the patient is stable especially with de novo disease [9], and preferable within 48 h of admission if possible [16].

Furthermore, lung and cardiac ultrasound facilitates the diagnostic workup of patients with acute respiratory failure, circulatory shock, or cardiac arrest [23].

Routine blood gas analysis is only required [9] if transcutaneous arterial oxygen saturation (SpO_2_) < 90% in ambient air. (Figure 2).

### 3.2. Therapeutic Management of HF and Precipitating Factors 

**Figure 3:**
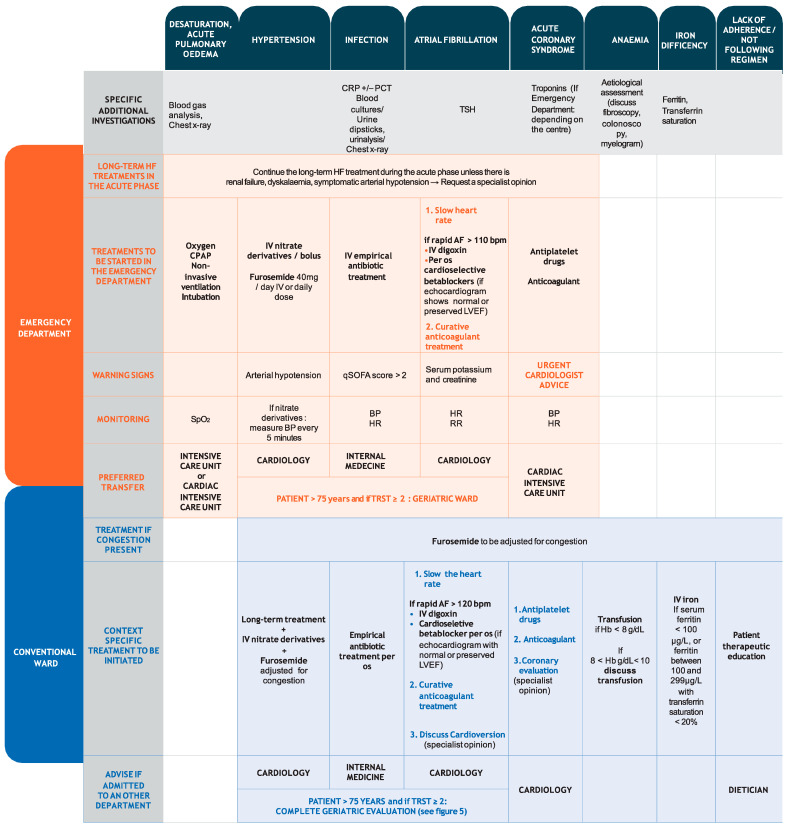
Therapeutic management. This figure was developed using expert consensus, guidelines and recommendations [9,16,24,29,30,31,32,33] and specific studies [34,35,36,37,38,39,40,41,42,43,44,45,46,47].

Long-term HF treatments must be continued throughout the acute phase. These are based on loop diuretics, usually intravenous, adjusting the dose to the clinical response. In patients with HFrEF, continuing angiotensin-converting enzyme inhibitors (ACE-I) or angiotensin receptor blockers (ARB) and beta-blockers (BB) is recommended with doses adjusted according to the haemodynamic state. Treating precipitating factors is of paramount importance. If renal failure, dyskalemia or symptomatic hypotension occur, a specialist’s opinion should be sought. (Figure 3).

#### 3.2.1. Thromboembolism

In the acute phase, thromboembolism prophylaxis should be considered for all patients with HF or AHF, unless the patient is already taking anticoagulants or has a contraindication. The European Society of Cardiology (ESC) recommends an oral anticoagulant to prevent thromboembolism for all patients with paroxysmal or persistent/permanent AF and a CHA2DS2-VASc score ≥2 (men) or ≥3 (women), irrespective of whether a rate or rhythm management strategy is used (including after successful cardioversion). Anticoagulants should be considered if the CHA2DS2-VASc score is ≥1 (men) or ≥2 (women) [16]. 

#### 3.2.2. Hypoxia

If SpO_2_ < 90% oxygen therapy should be considered and initiated in the Emergency Department (ED) [9]. Blood gas analysis (pH, acid-base balance, oxygen, and carbon dioxide partial pressure tension, and possibly lactate) should be evaluated, especially in patients with pulmonary embolism (PE) or previous history of chronic obstructive pulmonary disease (COPD). Although arterial blood is preferable, especially for patients with cardiogenic shock, venous blood measurements can be used [9,16]. 

If respiratory distress occurs (respiratory rate > 25 breaths/min, SpO_2_ < 90%, hypercapnia and pH < 7.35) [9], non-invasive positive pressure ventilation (bilevel positive airway pressure, continuous positive airway pressure) should be considered. Early ventilation improves respiratory distress, reduces the need for mechanical endotracheal intubation and may reduce mortality [16]. BP can decrease with non-invasive ventilation so close monitoring in hypotensive patients is required [16]. As intubation is often problematic in older patients (aged > 75 years), non-invasive ventilation is a valuable solution, having a positive effect on the heart by decreasing afterload and increasing preload [16].

If respiratory failure is causing hypoxaemia (partial oxygen pressure (PaO_2_) < 60 mmHg (8.0 kPa)), hypercapnia (PaCO_2_ > 50 mmHg (6.65 kPa)) and acidosis (pH < 7.35) and non-invasive management is unsuccessful, intubation is recommended [16]. 

Lung ultrasound can be a valuable tool to guide positive end-expiratory pressure settings in mechanically ventilated patients and can be used to assess treatment efficacy, monitor respiratory disorder, and guide ventilation weaning. It also facilitates early detection and management of mechanical ventilation-associated respiratory complications [23]. Any patient with hypoxia or PE must be transferred to intensive or cardiac intensive care (CICU). 

#### 3.2.3. Hypertension

Furosemide treatment is only indicated in patients who have HF with signs and symptoms of congestion. If not given prior to admission, 20–40 mg intravenous furosemide should be administered initially to patients not taking an oral diuretic. For patients taking chronic diuretic treatment, the initial intravenous furosemide dose should be at least the same as the usual oral dose [9,16,34]. If volume overload is present, the IV diuretic dose should be adjusted according to the HF type (lower dose for de novo and higher for decompensated CHF) [9]. Ideally, the diuretic dose should follow guidelines but in practice, 80 mg furosemide can be given if required. Additionally, a prospective, observational study in patients with AHF found that administering IV furosemide in the ED, within 60 min of onset, lowered in-hospital mortality [35]. 

When systolic BP is normal to high (>110 mmHg), intravenous vasodilator therapy can be used for symptomatic relief [9]. IV nitrates can be given in 3 mg boluses every five minutes. After one hour of bolus titration, treatment is changed to a continuous infusion with an hourly dose of at least half the total given during the first hour. BP must be monitored every five minutes during titration and then hourly. If the BP drops below 100 mmHg, discontinue nitrate treatment. Alternatively, sublingual nitrates may be considered [34]. These patients should be transferred to a specialist cardiology unit. 

#### 3.2.4. Infection

Following specific investigations (Figure 3), antibiotic treatment, usually amoxicillin and clavulanic acid, should be started according to local guidelines if at least two of the following findings are present: temperature > 38 °C, leucocytes > 12,000 G/L, radiological signs of lower respiratory tract infection or elevated C-reactive protein or procalcitonin [34]. 

The quick Sequential Organ Failure Assessment (qSOFA), a 0–3-point score, is a validated tool to detect sepsis quickly. One point is given for each sign; systolic hypotension (<100 mmHg), tachypnoea (>22 bpm) or altered mentation [36]. Scoring > 2 is a warning sign. The use of qSOFA in the ED has a statistically greater predictive validity for in-hospital mortality than SOFA and SIRS (systemic inflammatory response syndrome) [36].

Community-acquired pneumonia (CAP) is a common precipitating factor for HF decompensation [48]. CAP frequently affects very old adults (>80 years) and presentation is uncommon so diagnosis can be difficult. Currently, there are no robust, validated AHF-CAP severity classification tools or specific management recommendations in the ED for these critically ill patients. Management should be guided by baseline characteristics, clinical presentation, and risk factors for multidrug-resistant pathogens [37], and treatment started immediately whilst in the ED [38]. The Infectious Diseases Society of America (IDSA)/American Thoracic Society (ATS) guidelines for CAP antimicrobial regimens are relevant for older patients and should be used accordingly [49]. Empirical antibiotic treatment is advised while considering the most common pathogens and their associated severity. Ideally, antibiotics should be started within 4 h of diagnosis and efficacy evaluated after 48–72 h. Amoxicillin is the first choice for patients with suspected *S. pneumoniae* but this can be changed to a macrolide or amoxicillin/clavulanic acid if atypical bacteria are suspected or the patient is frail or has comorbidities. For patients in the ICU, IV third-generation cephalosporin with a macrolide or anti-pneumococcal fluoroquinolone is recommended [50]. Other regimens are indicated if methicillin-resistant *Staphylococcus aureus* or *Pseudomonas aeruginosa* is a concern [38].

There is little evidence that procalcitonin-guided antibiotic prescription is effective so should be avoided. 

It is important to monitor BP, HR and transfer patients to internal medicine. 

#### 3.2.5. Atrial Fibrillation

It may be difficult to differentiate between AF and HF symptoms, especially in HFpEF but the HFA-pEFF score can help with diagnosis [51]. When AF occurs, identifying potentially correctable causes such as hypo/hyperthyroidism, uncontrolled hypertension, electrolyte disorders, and mitral valve disease, and precipitating factors such as recent surgery, chest infection, exacerbation of COPD/asthma, acute MI, and excess alcohol consumption is essential [16]. The need for an anticoagulant regimen is based on the stroke risk assessment and risk/benefit balance [24]. Ventricular rates should be checked [16] to maintain HR under 110 bpm [34,52]. Oral beta-blockers can be started for rate control if there are no distressing HF symptoms. If the patient has marked congestion but few symptoms at rest, initial treatment with oral or IV digoxin is preferable [16].

Patients with elevated troponin should be admitted to the CICU [34]. Warning signs include abnormal potassium and creatinine. Heart rate and respiratory rate should be monitored, and patients transferred to cardiology.

#### 3.2.6. Acute Coronary Syndrome

Dual antiplatelet therapy must be administered and the patient transferred to the CICU if at least two of the following are present: chest pain, ischaemic signs on an ECG, elevated troponin concentration or change in troponin concentration. Coronary angiography should be performed as recommended by international guidelines [29,30,34].

Renal dysfunction often occurs with chronic, acute, or decompensating HF, and should be managed with both a cardiologist and a geriatrician. Previous creatine levels should be carefully checked to differentiate between an acute episode and chronic exacerbation [53]. In hypervolemic patients, congestion drives renal dysfunction. Systemic venous pressure increases renal interstitial pressure which reduces glomerular filtration rate (GFR). Appropriate decongestion using diuretics improves GFR [54]. Renal function decline is not an indication to reduce the diuretic dose [39]. 

#### 3.2.7. Cardiac Cachexia

Cardiac cachexia is defined as nonintentional and non-oedematous weight loss ≥ 6% of total body weight, occurring over the previous 6–12 months and may affect around 30% of HF patients over the age of 80. It is characterised by sarcopenia, osteopenia and loss of fat tissue.

Cardiac cachexia increases the risk of autonomy loss and mortality (50% mortality at 18 months). Detecting cardiac cachexia early is critical to begin nutritional support and appropriate adapted physical activity [16].

## 4. Stabilisation and Management Phase 

### 4.1. Congestion

The vast majority of Acute heart failure (AHF) episodes also have congestion symptoms (including breathlessness, orthopnoea, fatigue, oedema, and nocturnal dyspnoea) and signs (including elevated jugular venous pressure, third heart sound, cardiomegaly and hepatojugular reflux) [16,39,53,55]. A complete clinical congestion assessment should be performed using a multimodal approach. The EVEREST clinical score is currently the most evidence-based and appropriate routine score. Measuring plasma volume and haemoconcentration can be useful as can chest X-ray, echocardiography, and lung ultrasounds [53]. Clinical congestion parameters are important and have a prognostic value [56]. Echocardiographic assessment of inferior vena cava diameter is simple and may provide an objective, quantifiable measurement of right atrial pressure. It also provides prognostic information since increasing inferior vena cava diameter is associated with a worse prognosis [57]. Multimodal congestion assessment during admission, treatment and discharge could be useful and ideally, optimal decongestion should be achieved prior to discharge [53] (Figure 2 and Figure 3).

### 4.2. Management of Precipitating Factors

#### 4.2.1. Infection

For critically ill, elderly patients with community acquired pneumonia (CAP) not requiring invasive procedures and for whom intensive care admission has questionable benefit, hospitalisation in intermediate care may be appropriate [37]. 

The IDSA/ATS guidelines for CAP antimicrobial regimens should be followed and are relevant for older people [49]. However, watch for medication-related adverse events, cardiovascular events, and exacerbation of comorbidities in this population [38,58].

Once haemodynamically stable, treatment should be changed to oral antibiotics and discharge considered [38]. A prospective, randomised, non-inferiority trial showed that IDSA/ATS recommendations for short, five-day treatment duration are safe in hospitalised CAP patients [40] (Figure 2 and Figure 3).

#### 4.2.2. Atrial Fibrillation and Acute Coronary Syndrome

The treatment strategy involves either reducing heart rate, adding an anticoagulant [16] or considering cardioversion. Anticoagulation should be used in patients with HF and AF (including elderly patients) and the benefit and risk evaluated using the CHA2DS2-VASc and HAS-BLED scores. The CHA2DS2-VASc score emphasises the importance of increased age in thromboembolic risk evaluations [59]. Non-vitamin K antagonist oral anticoagulants (NOAC) are preferred to vitamin K antagonists in HF patients with non-valvular heart failure (HF). The dose must be adjusted according to age (>80 years), renal function and weight. However, NOACs are contraindicated in patients with mechanical valves or moderate mitral valve stenosis [16]. Beta Blockerss, digoxin, diltiazem and verapamil or combination therapy can be used for rate control. BBs and/or digoxin are recommended rate-control treatments in AF patients with HFrEF. In the elderly, rate control is first-line therapy. 

Cardioversion, whether electrical or pharmaceutical, is related to serious side effects in the elderly [52,59]. Further practical information is illustrated in Figure 3 and full details can be found in the current guidelines. 

Percutaneous and surgical revascularisation are complementary approaches for symptomatic relief of angina in HFpEF, but whether these interventions improve outcomes is not entirely clear and depends on frailty status, not necessarily age. Recent ESC guidelines on myocardial revascularisation recommended coronary artery bypass grafting [16]. Randomised controlled trials (RCT) including patients with HFrEF revealed that revascularisation did not reduce overall mortality, even in subgroups of patients with angina or myocardial ischaemia, but further analysis suggested a reduction in sudden deaths [16] (Figure 2 and Figure 3).

#### 4.2.3. Anaemia and Iron Deficiency

Anaemia is defined as a haemoglobin concentration < 13.0 g/dL in men and 12.0 g/dL in women and is more common in older patients. Whenever anaemia is found, a diagnostic workup is essential (e.g., occult blood loss, iron deficiency (ID), blood dyscrasias) however the cause is often not discovered [16]. If haemoglobin is <8 g/dL, provide a transfusion, and if between 8 and 10 g/dL, discuss providing a transfusion. Anaemia and ID are independent risk factors for adverse outcomes in CHF and acute decompensated HF [41] with anaemia being an independent predictor of mortality [42].

ID is a common, often overlooked, and undertreated comorbidity. It is defined as serum ferritin < 100 μg/L, or ferritin between 100 and 299 μg/L with transferrin saturation < 20% [16]. Iron status screening should be performed in all CHF patients [16] because, even in the absence of anaemia, ID aggravates the underlying disease and negatively impacts clinical outcomes and quality of life [44]. The ESC recommends IV iron for symptomatic HFrEF patients with ID [16]. Recently, ferric carboxymaltose has been shown to be safe and reduces HF hospitalisation risk, with no apparent effect on the risk of cardiovascular death in patients stabilised after an episode of AHF with ID and LVEF < 50% [60] (Figure 2 and Figure 3).

### 4.3. HF Management 

Most therapies reverse or slow cardiac and peripheral dysfunction to improve prognosis and symptoms and reduce mortality and morbidity. For hospitalised patients, reducing hospitalisation duration and subsequent readmissions, preventing organ system damage, and managing comorbidities are additional goals.

Neurohormonal modulators (Renin-Angiotensin-Aldosterone System (RAAS) inhibitors, mineralocorticoid receptor antagonists (MRA)) and BBs significantly improve HFrEF-related morbidity and mortality. MRAs should be used with caution in patients over 80 years of age. However, considering the benefits provided by sacubitril/valsartan, the 2016 ESC guidelines recommended replacing ACE-I/ARBs with an angiotensin receptor-neprilysin inhibitor (ARNI) to reduce the risk of HF hospitalisation and death in symptomatic, ambulatory HFrEF patients (NYHA class II to IV) despite optimal medical treatment [61]. However, if symptoms persist, the ACE-I can be replaced with a neprilysin inhibitor. The ACE-I 36 should be discontinued 36 h before introducing the sacubitril/valsartan combination the dose titrated according to blood pressure tolerance, renal function and serum potassium should be monitored [6,22]. No RCTs have been conducted in the elderly HF population but all RCTs conducted in HFrEF patients have shown the beneficial effects of ACE-I/ARBs [62], BBs [63], MRAs [64] and sacubitril/valsartan [65].

The recent studies of sodium-glucose co-transporter-2 inhibition showed a beneficial effect when HFrEF (LVEF < 40%) patients were already on stable optimal medical treatment [66,67].

Decompensated HFrEF patients should have their diuretic dose adjusted for congestion, and BBs, MRA, ACE-I and RAAS inhibitors titrated if required. Preventive low molecular weight heparin for 5 days and ID correction can also be considered. [68]

More than 50% of HF cases are HFpEF. In these cases, adjust the diuretic dose and optimise hypertensive, antiarrhythmic, and anti-ischaemic treatments accordingly. Importantly, investigate HFpEF aetiology. Recently, transthyretin amyloidosis (ATTR), has been found in around 13% of patients with HFpEF [69], 16% of patients with aortic stenosis [70] and some elderly men with carpal tunnel syndrome, and can be easily diagnosed using bone scintigraphy. ATTR is a clinical disorder characterised by misfolded proteins that form insoluble amyloid fibrils which aggregate in cardiac and other tissues. Tafamidis, a new treatment for these patients improves survival [71].

At this stage, patient therapeutic education, dietitian input, and cardiovascular rehabilitation should be considered [72]. 

Management should always focus on the four cornerstones of regular Weighing, treatment Adherence, healthy Salt diet (<5 g/day) [73] and Physical exercise (WASP). 

Current international guidelines recommend specific measures for preventing CAP [37]. One of the most effective preventive methods in patients aged 65 years and over is through vaccination for influenza and *S. pneumoniae* [38].

Hypotension is common in HFrEF and may limit treatment titration. If symptomatic or severe hypotension (systolic BP < 90 mmHg) occurs, firstly decrease BP-lowering drugs not indicated for HFrEF and loop diuretics if there are no signs of congestion, otherwise consult a cardiologist [73].

If renal function worsens, medication may be adapted according to clinical scenarios (congestion, dehydration, hypotension, or hyperkalaemia) [74]. 

**Figure 4:**
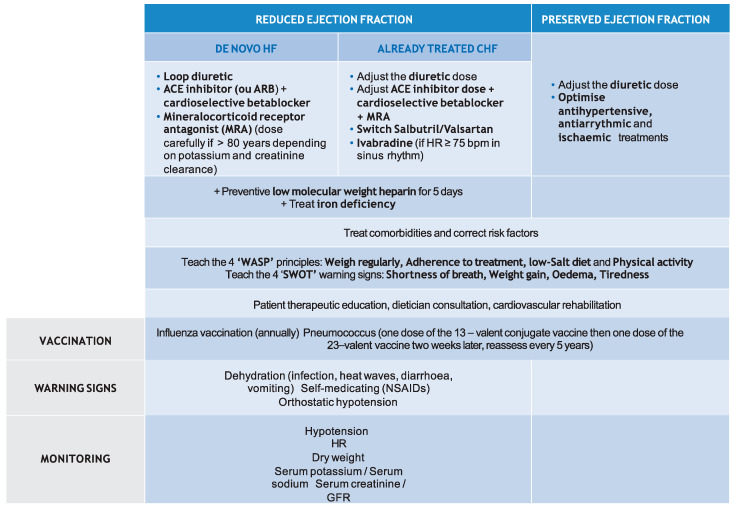
Therapeutic management of heart failure. This figure was developed using expert consensus, guidelines and recommendations [6,7,14,16,33,74,75,76,77,78,79,80,81,82,83,84,85].

Monitoring of all HF patients includes BP, HR, dry weight, serum potassium, sodium, and creatinine, and GFR. (Figure 4).

### 4.4. Geriatric Syndrome Management 

**Figure 5:**
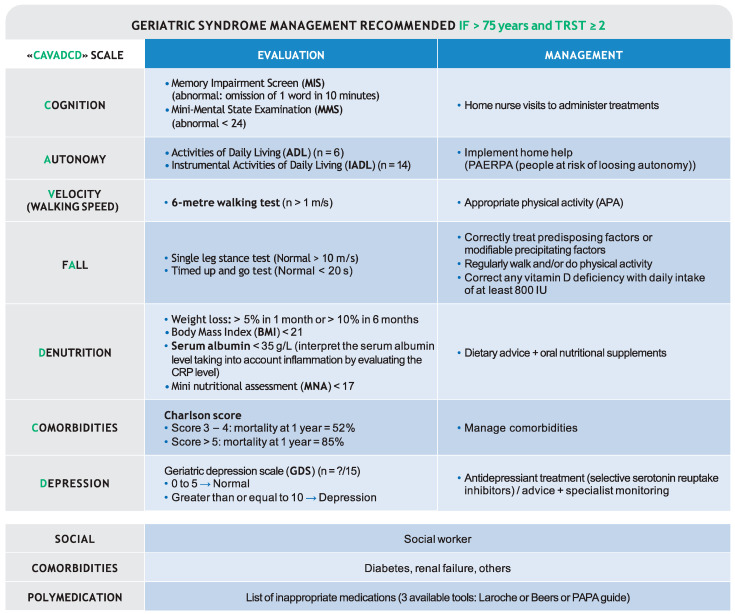
Geriatric syndrome management. This figure was developed using expert consensus, guidelines and recommendations [75,76,77,78,79,80,81,82,83,84], and specific studies [85].

Older patients requiring a geriatric evaluation must be identified. A prospective, national, multicentre study showed the triage risk screening tool (TRST) is useful [45]. The TRST measures five indicators: cognitive disorders, difficulty walking/moving/falls, polymedication, history of hospitalisation in the last 90 days or admission to the ED in the last 30 days [86] and loss of autonomy. It should be performed within 48 h of admission for any patient aged > 75 years. A score > 2 indicates geriatric referral is needed. The authors suggest using the CAVADCD scale, with warning signs of looming death (Cognition, Autonomy, Velocity, fAlls, Denutrition, Comorbidities, Depression) as the basis for a comprehensive geriatric evaluation. (see Figure 5).

Cognition is best measured with the mini-mental state examination the shorter Memory Impairment Screen must be used to identify cognitive impairment risk (Figure 5) [87].

Autonomy can be evaluated using the Activities of Daily Living (normal = 6) and the Instrumental Activities of Daily Living (normal = 14). Different Management methods are available. PAERPA (people at risk of loss of autonomy) personalised health plans can be used to analyse the identified problems, taking the patient’s expectations into consideration. Equal importance is given to medical, psychological, and social aspects [76,77]. These methods aim to preserve the autonomy of older people and ensure they receive good health care at the correct place, time, and price. The five key elements to consider are increased home support, improved coordination of caregivers and procedures, secure hospital discharge, avoiding unnecessary hospitalisations and improved medication use [78].

Velocity (walking speed) is assessed using the 6-metre timed walk (normal > 1 m/s). Any problems should be managed with appropriate physical exercise and vitamin D deficiency correction. 

If falls have been reported, orthostatic hypotension must be investigated (decrease of 20 mmHg for systolic BP or 10 mmHg diastolic BP within 3 min of standing) and management should include revising prescription medications for falls. Predisposing or modifiable precipitating factors must be corrected or treated. Dietary calcium should be between 1 and 1.5 g/day and any vitamin D deficiency corrected with a daily intake of at least 800 IU [27]. 

Denutrition is diagnosed if one or several of the following criteria are present [79]: weight loss: >5% in 1 month or >10% in 6 months, BMI: <21, serum albumin <35 g/L (interpret the serum albumin level taking into account inflammation by evaluating the CRP level) and mini nutritional assessment (MNA) < 17. Management strategies include dietary advice, enriched food and oral nutritional supplements and the patient must be monitored closely once they are stable. 

Comorbidities and life expectancy may be assessed using the Charlson score. A score of 3–4 is associated with a 52% mortality risk at 1 year, a score ≥ 5 is associated with an 85% mortality risk at 1 year [88]. 

Depression is strongly related to symptoms and isolation but sadly is insufficiently diagnosed. The geriatric depression scale (GDS) is a useful assessment scale, including 15 questions. A score of 0–4 = normal, 5–8 = mild depression, 9–11 = moderate depression, 12–15 = severe depression. Management includes antidepressant treatment with selective serotonin reuptake inhibitors, advice, and specialist monitoring [80,81]. 

Frailty is a syndrome defined by decreased functional reserves, which significantly increases mortality risk and occurs in 45% of older HF patients [89], fueling a vicious exacerbation cycle with both cardiovascular and non-cardiovascular comorbidities [22]. Evaluating frailty is multidimensional and multiprofessional and can be conducted in any setting. The Fried assessment criteria define frailty by the presence of at least three of the following criteria: unintentional weight loss (more than 4.5 kg in one year), extreme exhaustion, slow walking speed (speed < 1 m/s), muscle weakness (time to get up from a chair 5 times in a row without using arms > 12 s) and sedentariness. 

Multiprofessional frailty management can reduce the risks of dependency, hospitalisation, and nursing home admission. The management process is initiated and coordinated by the treating clinician and may include a personalised health plan with support from the patient and other primary health care professionals with geriatric expertise.

Polymedication is common in elderly patients. However, physiological changes, comorbidities and certain pathologies are associated with potential undesirable effects. Drugs with an unfavourable benefit to risk ratios such as lipid-lowering therapies like statins [90], renal toxic therapies such as NSAIDs, and corticosteroids may be inappropriate in geriatric patients when other, safer therapies are available. Additionally, concomitant medications for comorbidities should be minimised, antidepressants should be avoided, and patients with diabetes should be reminded of glycaemic targets to avoid overmedication. 

Several tools include Laroche [85], the American Geriatrics Society (AGS) Beers Criteria^®^ (AGS Beers Criteria^®^) [82] and the 42-page guide to good medical prescribing for people aged 75 years and over (PAPA guide) created by the French Society for Geriatrics and Gerontology (SFGG) and the National Professional Geriatrics Council (CNP).

### 4.5. Patient Discharge: A Transitional Care Intervention

Patient discharge planning should start when the patient is stable and euvolemic, and precipitating factors have been treated [16]. Transitional Care Interventions such as PRADO, assist the continuity of care after discharge. Effective discharge interventions include detailed discharge summaries and early prearranged and structured physician follow-up [91]. Older, frail HF patients must be monitored, and any reversible cardiovascular and non-cardiovascular causes of frailty should be identified and addressed [16]. Referral to the geriatric team for specialist care must be considered for follow-up and patient and carer support [7]. 

Comorbidities must also be diagnosed and treated e.g., COPD, renal failure, anaemia, malnutrition, diabetes and depression [7]. Multidisciplinary meetings facilitate creating a personalised health plan and an end-of-life plan with both the patient and their next of kin [22]. 

To guarantee continuity of care, post-discharge management must be multiprofessional [31] involving the generalist, cardiologist, and geriatrician monitoring if needed. Coordination with other primary caregivers and non-hospital services is also essential [7]. Home visits by specialist HF nurses reduce readmission and mortality [31]. The patient should consult their GP the week after discharge and the cardiologist 1 week to 2 months after discharge [7]. Further consultations may be required depending on progress [7]. Early physician follow-up reduces the 30-day readmission incidence [16].

The main follow-up aims include continuing treatment titration, implementing treatment education to monitor symptoms and raise alerts, ensuring rapid access to care in case of an alert and prescribing regular physical activity appropriate for the patient [31].

Patient medication must be carefully reviewed to optimise HF medication [16]. For long-term HF medications, a titration regimen should be anticipated or suggested. If LVEF < 35% and QRS > 130 ms, a specialist opinion for resynchronisation should be sought. Polypharmacy should be reduced and any medication without an immediate effect on symptoms should be discontinued. Reviewing diuretic therapy timing and dose helps reduce the incontinence risk [16]. 

Giving patient information and education about self-care improves outcomes [16,73]. Education should also aim to help the patient and their family understand the reasons for decompensation, adhere to treatments and prevent recurrence [7]. Teaching patients about SWOT (4 alert signs) and WASP is particularly important.

Telemedicine (especially telemonitoring) can be a useful and efficient follow-up tool. It ensures warning signs are picked up quickly and can facilitate ambulatory management [22]. 

## 5. Conclusions

This review aims to improve care and promote an updated system for HF care in a hospital environment according to the latest available evidence. HF management remains suboptimal with frequent patient readmissions and varying outcomes. Promptly recognising HF signs, frailty and comorbidities with validated checklists reduce therapeutic inertia. Improving access to cardiology consultations with multidisciplinary, in-hospital and patient care pathways improve survival. Hospitalisation is an opportune time to optimise HF treatment and educate patients about self-care, adherence, and lifestyle.

## Data Availability

Not available.
